# Hard to reach? Methodological challenges researching vulnerable, gang‐involved, young people

**DOI:** 10.1111/hex.14092

**Published:** 2024-06-04

**Authors:** Rhiannon Barker, Chris Bonell, G. J. Melendez‐Torres

**Affiliations:** ^1^ Public Health Environments and Society London School of Hygiene and Tropical Medicine London UK; ^2^ School for Public Health Environments Research, Medical School University of Exeter Exeter UK

**Keywords:** ethics, hard to reach, qualitative research, research ethics committee, vulnerable

## Abstract

**Introduction:**

Research with young people (YP) is ethically challenging and bound in a complex maze of issues relating to power, voice and representation. Such sensitivities mean that the challenges raised in researching marginalised YP are often hard to navigate. This paper reports on research carried out with YP to explore links between mental health, school exclusion and involvement in criminal gangs. It aims to provide a practical guide to negotiating some of the methodological and ethical challenges experienced.

**Method:**

In‐depth interviews conducted with 28 YP (aged 14–24 years) who were gang involved or seen to be at risk of gang involvement. Research was conducted in youth clubs, alternative provision and youth justice settings.

**Results: Observations/Reflections:**

We reflect on how navigating ethics can create barriers to involving YP as primary informants in research. We consider why it is important to overcome these hurdles and how public engagement work with recognised gatekeepers and the use of creative interview methods can facilitate meaningful encounters, where YP feel able to share valuable insights into their lives.

**Conclusion:**

Alongside a number of specific learning points, the paper reflects on theories behind research with YP, including the need for recognition of power imbalances and reflexivity. It concludes with thoughts on the practical realities of achieving meaningful participation or an ‘authentic voice’ with marginalised groups and the importance of this in informing policy and practice.

**Patient or Public Contribution:**

The focus of this work was to collect experiences of YP who are recognised as gang‐involved or at risk of being so, with a view to informing health and education policies. The scoping study for the project involved extensive public engagement work with YP exploring and trialling suitable methods of accessing, recruiting and ultimately interviewing this target group. This is central to the discussion within the body of the paper.

## 1 INTRODUCTION

In this paper, we explore methodological challenges in conducting qualitative research on sensitive topics with vulnerable young people (YP). Accessing groups deemed vulnerable or whose voices are seldom heard for research purposes may require the use of methods and tools that go beyond conventional qualitative techniques. While our primary method was face‐to‐face in‐depth interviews, we draw on more transformative research frameworks, built around methods that focus on the possibilities of supporting new discovery and paradigm shifts,^1^ using community based and participative methods. We highlight how, while no one method is a panacea for working with marginalised YP, there do appear to be techniques which help maximise benefits for participants, through building trust, engagement and rapport, as well as benefitting the quality and integrity of research outputs.

The research on which this paper is based investigated mechanisms contributing to gang involvement in YP aged 14–24 years. The work was undertaken following the pandemic at a time when concerns were raised about the numbers of marginalised YP becoming involved in criminal gangs, groomed by gang leaders and not accessible by formal support services.^2^ The central questions looked at the assignation of meaning and agency during states of transition into gangs with a particular focus on the intersection between mental health, school exclusion and gang involvement. Sensitivities encountered during the work were manifold including: the exploration of areas which may be hard to talk about publicly due to their illegal nature; stigma connected with exploration of subcultures touching on race, ethnicity and violence and issues which may generate an emotional response due to harms suffered.

The project comprised:

1. Public involvement and engagement including consultation with professional stakeholders and YP. A steering group (education and charity professionals working with marginalised YP, civil servants and professional mentors with lived experience of gang‐involvement), to guide research and help disseminate and embed outcomes.
2. In‐depth interviews with YP who have experience of criminal gangs and related professionals.

This paper focuses on methods used to access and interview YP. The majority of participants were school‐excluded, had mental health issues, criminal convictions and a range of complex family challenges. Providing a channel for the voice of such marginalised YP is key to building appropriate services, yet accessing vulnerable YP is problematic.^3^, ^4^, ^5^ The majority of participants tended not to access community‐based formal networks, meaning they are harder for researchers and professional stakeholders to reach. Even when YP are reached and give consent for interview, YP with experience of the criminal justice system are often mistrustful, fear reprisals or are simply unable or unwilling to share personal information.^3^, ^4^

We begin with looking at some of the theory implicated in this work and then consider the hurdles encountered at each stage of the research process—from inception to completion covering: (i) ethics; (ii) public engagement; (iii) accessing gatekeepers; (iv) approaching YP; (v) Interview methods and (vi) response of YP and continued engagement with outcomes. The paper concludes by discussing why it is important to persist in finding ways to access the most marginalised.

## 2 BACKGROUND

The notion of ‘vulnerability’, both attributed to children per se and more particularly to specific high‐risk groups of children, has meant that, in relation to ethical considerations, fear of doing harm has trumped the principles of ‘beneficence’.^6^ The level of agency and cognitive capacity YP possess as they grow older determines the approach that researchers take when working with them.^7^, ^8^

Social researchers guided by interpretivist qualitative research theory, have stressed the importance of engaging YP using person‐centred methods where they are encouraged and enabled to contribute to the process of constructing meaning of their lives and actions.^7^ This paradigm, while it maintains the ethical imperative of protection from harm, foregrounds, the human rights discourse of entitlement and obligation as intoned in the Convention on the Rights of the Child.^9^ Children are increasingly viewed as social actors whose knowledge and views should be both investigated and considered^9^, ^10^ and success in this regard is reliant on finding ways to engage them in research.

### 2.1 Power and voice

YP traditionally have low levels of agency and power in relation to decision making; their views on the structures around them and how these impact on their experiences are seldom sought. Yet power and voice influence and impact on methods of research and there is a growing body of work exploring these issues.^11^ Young children have rights; they are agents and active constructors of their social worlds, recognising and finding creative ways of channelling this agency has the potential to modify power differentials.^11^

### 2.2 Ethical considerations of work with marginalised YP

Ethical considerations reside at the core of all qualitative work with YP, particularly those deemed vulnerable or marginalised. The potential for harms including coercion, exploitation and personal damage, may be more pronounced in qualitative research, where the boundaries are fluid and the content of the research more open to interpretation, than in quantitative studies.^12^, ^13^ At the same time, there are recognised benefits with YP increasingly seen to have a central role in helping to shape policy and practice. As a rule, it appears that, while meaningful participation in research can raise self‐esteem and help YP feel they can make positive contributions to society,^14^ the potential for harm, particularly from the dangers of distress caused by revisiting past trauma, should be carefully monitored.^15^ Concerns of coercive or harmful practice are raised particularly where participants are considered ‘vulnerable’ and this in turn has been shown to limit opportunities of these groups for self‐determination and input into decision making. One proposed solution here is to consider the person in context, so vulnerability is viewed not solely as an individual trait but as something both situational and relational.^6^

For public health research, not involving investigational medical products, ethical regulations are governed by common law. Pertinent is the Gillick ruling relating to healthcare decisions^16^ which states that, regardless of their age, if children are able to understand the nature and consequences of participation, and to retain, express and communicate their decision—they are deemed competent and should, on this basis, be allowed to give their own informed consent.^17^

## 3 METHODS

### 3.1 Research design

This was an interpretive study, based on the premise that understanding of the world is built through social constructions manifest in language and symbolism and developed through human experiences and social contexts.^18^ In talking to YP, our intention was not to hold a mirror up to the ‘real world’ but to provide a partial picture of their own cultural and psychological interpretations of their experiences, motivations and beliefs.

### 3.2 Ethics process

The UN Convention on the Rights of the Child ^9^ states that children be given a voice at all levels of decision making. Historically in the United Kindom, research with YP has either involved, as a prerequisite, the signed consent of parents or the right to opt out. We argued, in this case, that the necessity for such permission would compromise the integrity of the research. Previous research has shown how such stipulations may reduce sample size and skew the final sample, meaning that the data are no longer representative of the target population.^17^ While this may not necessarily impact in the same way on qualitative work there was, we concluded, a valid concern that such action would hamper our ability to get a purposively diverse sample. Written consent was therefore obtained from YP, in addition to fulfilling ethical guidelines stipulated by individual partner organisations/settings. Ethics permission was received from the London School of Hygiene and Tropical Medicine ethics committee in November 2022 (reference: 26873).

While project information sheets were handed to participants before the interviews, in reality there was little inclination to digest over two pages of printed text. Acknowledging this, a thorough process of oral briefing was undertaken including the aims of the research, limits to confidentiality in the event of safeguarding concerns and reiteration that participation was voluntary.

### 3.3 Recruiting

Consultation with researchers during the scoping phase, who had conducted research with criminally involved YP, suggested that recruitment for this project was going to be challenging. Both statutory and voluntary organisations working with vulnerable YP are reportedly nervous about involvement in research which isn't directly related to evaluation of their own work, with anxieties voiced about data leaks and information breaches.^20^, ^21^ Objections were seen to be largely technical and legal rather than moral. These concerns were borne out and recruitment of YP to this project was slow and laboured. Numerous professionals were helpful and interested, but when it came to supporting with access, this did not translate into successful recruitment. Difficulties of access and recruitment appeared to reflect a number of factors: mistrust; itinerant lifestyles; absence of target group from mainstream services and nervousness on the part of gatekeepers working with vulnerable YP.

### 3.4 Gatekeepers

All YP were approached through gatekeepers, many of whom had built long‐term trusting relationships which were valued on both sides. A number of these gatekeepers were supportive of facilitating access in a sensitive fashion, built around the needs and desires of the participants. Indeed, it was these gatekeepers who extended their existing ‘bond of trust’ without which this research would not have been possible.

Most individuals and organisations contacted (both statutory and third‐sector), for a range of apparently valid reasons, were unable to support the research. Stated reasons for this included: being overburdened with competing pressures; the perceived burdens of General Data Protection Regulation compliance; concerns about having their service judged; research not being viewed as a priority; or worries about safety. There is certainly a degree of nervousness about regulatory requirements in supporting external research, though some of these might be overcome by clarifying the legal context. Challenges such as these can result in researchers being reluctant to carry out primary research, with many opting for older (adult) participants or using professionals/parents as a proxy.

Recruitment ultimately was driven by pragmatics, a summary of the process is shown in Table 1.

**Table 1:** Routes to recruitment.

Contacts made where recruitment was unsuccessful		
Types of agencies contacted	What did the process involve?	Recruitment?
30 Named contacts in local authorities and YOTs (family protection, serious violence, youth justice teams)	Numerous emails exchanged and a few exploratory meetings in which R. B. spoke about and answered questions on the project. Organisations demonstrated very different governance requirements for ethics/GDPR.	1 London‐based local authority supported the research through providing contact to organisations they commission; youth clubs, therapeutic support, YOT.
15 Emails/phone calls to charities working to support gang involved young people	No response from the majority. Phone calls and emails exchanged with five organisations.	1 Charity working with gang involved YP in the south‐west supported recruitment.
8 Named contacts in schools and alternative provisions contacted	No response from majority. One school went through a number of regulatory checks but then withdrew. It was suggested that research with YP in schools relating to criminal behaviour may cause reputational damage with parents.	1 Liverpool‐based alternative provision supported recruitment.

Abbreviations: GDPR, General Data Protection Regulation; YOT, youth offending team; YP, young people.

### 3.5 Accessing YP and gaining trust

Once organisations and gatekeepers had come on board as research partners, potential participants were contacted in a number of ways:

1. In the majority of cases, gatekeepers gave the researcher (R. B.) access to settings (alternative provisions and late‐night youth club) where she was able to spend time ‘hanging out’, watching activities, playing snooker or eating with participants. This provided an opportunity for R. B. to talk about her work and ask people if they would like to participate. This method of direct face‐to‐face contact in a setting with which YP were familiar was the most effective method of recruitment.
2. A local authority youth justice team asked YP on their restorative justice team whether they would like to participate. If they agreed they were able to deduct the time spent in the interview from their court orders, which stipulate the number of hours of community or other service they are required to serve. The assumption was that the interview involved aspects of reflecting on criminal involvement and the process was viewed as beneficial by the youth offending team. A couple of YP agreed to be interviewed through the restorative justice programme. These interviews were conducted in the presence of their restorative justice worker.
3. Two YP were contacted through mentors working to support gang involved YP. One of these interviews was conducted in the presence of the YP's mentor.

Twenty‐nine YP were eventually recruited. A summary of basic demographics of the sample interviewed can be found in Table A1.

### 3.6 Interviewing

Consent was sought ahead of the interview (see Section 3.2) and was reestablished at various moments during the interview, particularly in instances where there were signs that YP were growing agitated, upset or becoming disengaged.

This research involved in‐depth interviews rather than an extended ethnographic study. Time with respondents was limited, building rapport extended over hours rather than days or weeks.^20^ Included within the principle that children have a ‘right to be properly researched’ is the imperative that methods used should be appropriate and accessible.^22^ Cognisant of the importance of this, as part of the scoping for this project, public engagement work was conducted exploring appropriate interview techniques. Groups were run with mentors working with gang‐involved children, as well as a group of YP deemed to be at risk of school exclusion. During these sessions, techniques were trialled designed to reduce the intensity of the interaction between researcher and participant, and leaning on creative ways of tapping into embodied knowledge,^1^ refocusing away from the asking of questions towards building a more equal relational dialogue. Methods used borrowed from and overlapped with a number of different creative approaches^1^ and included arts based, embodied and participatory/transformative research.

Greene and Hill^23^ describe the process of researching children's experience as ‘fundamentally problematic’^23^ requiring insight into how the child interprets ‘self to self’, how they attempt to communicate this experience to others, as well as how others interpret and understand the reported experience. A growing literature on creative and participative approaches to interviewing children is suggesting ways to provide tools to meet some of these challenges, giving reign to a child's imagination while establishing an engaging and fun environment. Methods used have included creative writing, telling stories, drawing, painting, taking photos and making films.^1^, ^24^

Traditional ideas of the interview as ‘objective’ have been rebutted in recent years.^25^, ^26^, ^27^, ^28^ Factors including power differentials, gender, ethnicity, age and setting are all now seen to impact interview style and outcomes. Oakley,^26^ in a feminist critique, reframes the interview, challenging its presentation as a process where data are elicited in a largely one‐way exchange of information from respondent to researcher, in which participants are expected to tell the truth about their lives. To counter the risks of exploitative practice Oakely^26^ advocates for the building of rapport, at times involving the sharing stories and insights into one's own life.

**Figure B1:**
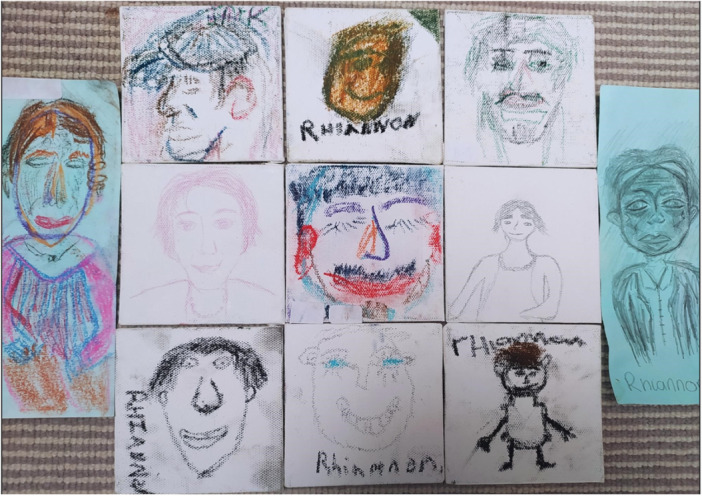
Collage of portraits drawn in interviews.

Alongside the influence of social, cultural and power dynamics the interview is affected by the setting. Practitioners working with vulnerable YP have emphasised the importance of creating a safe and welcoming environment.^5^, ^21^ Cognisant of this, we selected quiet safe areas where food and drink were offered. Using techniques drawn from previous qualitative research with marginalised and vulnerable YP, we provided crayons and big rolls of paper for doodling.^1^, ^11^, ^21^ In addition, at the beginning of each session, the interviewer asked participants if they would be prepared to paint her picture. The instructions were, using a small canvas provided, that the researcher and interviewee to do a quick sketch of each other. While a few declined the invitation, the majority, with a little persuasion, took up the challenge. The distraction, perhaps like jumping into cold water, diverted the YP from the tensions of the interview situation, and the fact that a similar task was being undertaken by both parties appeared levelling. The rudimentary artistic talents of the interviewer seemed to offer comfort and hilarity (see Figure B1). This provided a few minutes to settle into each other's company, before negotiating questions in the topic guide.

Following the warm‐up drawing exercise, word cards were used, representing the key themes to be covered in the topic guide: individual background (what do you like doing/what's important?); family and friends; experiences at school; looking back; gangs; mental health; and support services. At this stage, the YP was asked if there were any particular areas or issues they didn't want to talk about and were asked to push these cards away. More detail on specific challenges encountered during the interviews is discussed in Section 4. At times during the interview, to help visualise sequential narratives, drawing materials were used to create time‐lines to help jog memories and order events.

Interviews lasted between 30 and 90 min, and YP were rewarded for their time with £40 ‘JD Sports’ vouchers (acknowledging the time they had given up). Although most participants were interviewed just once, three YP had follow‐up sessions to sense test some of the findings and consult on their views about dissemination.

### 3.7 Additional data collection methods

In addition to interviews with YP, as far as possible, information was also collected from professionals working with them and from limited periods of observation of the YP within the youth club or alternative provision where they were interviewed. Information from professionals constituted a ‘pen picture’ covering basic demographics, family, education, mental health and offending history. Information collected from professionals was not proactively raised in interviews with YP but where information gathered from different sources did not align, this was noted. Field notes, recording moods, expressions, tone of voice and related characteristics, were kept from observation of children in both formal and informal settings and these were used as part of the analysis, contributing to the development of ‘bottom‐up’ theory.^29^ Pearlman and Michaels^30^ advise that the interpretations and perspectives of adults should be used with caution in helping to supplement the accounts of YP.

### 3.8 Reporting

Consolidated criteria for reporting qualitative research (COREQ) were used to assist explicit and comprehensive reporting. COREQ comprises a 26‐item checklist covering the domains of: (i) research team and reflexivity, (ii) study design, and (iii) data analysis and reporting.^31^

## 4 RESULTS: OBSERVATIONS/REFLECTIONS

### 4.1 Impact of setting and presence of professionals on interview content

The interview setting, together with the way the YP viewed the researcher and understood their reasons for participating, undoubtedly impacted the degree of openness and willingness of participants to fully engage. Most interviews were conducted with the researcher and YP alone in private rooms in settings with which YP were familiar—although (for the safety both of participant and the researcher) stakeholders remained in the building. In instances when it was suggested the young person may prefer to have the gatekeeper present, they were given this option, though in all cases they opted to be on their own. In the local authority youth‐justice team, the two interviews followed a different format; for the majority of the interview, the officer responsible for restorative justice scheme was present. Although it appeared that the relationships between the YP and their worker were positive and empathetic, these interviews felt more stilted. There was a sense of a greater power imbalance: while the YP had agreed to the process, it was nonetheless conducted within the framework of their enacting the various stipulations of their court order and, understandably perhaps, the setting didn't have the same relaxed atmosphere as a youth club or alternative provision. In a third case, a YP was interviewed with a mentor present in a private cubicle of a café, and in this case, the mentor appeared to give the YP confidence, prompting him at times to share anecdotes.

### 4.2 Participant response to interviews

Marginalised YP often present with high levels of mistrust to those in authority.^32^, ^33^ Amongst those with experience of the criminal‐justice system and particularly gang involvement, antagonism towards ‘snitching’ and the intense fear of retribution are heightened and can be problematic in building the necessary trust to conduct good qualitative research. A qualitative study working with ‘drug market actors’ found that, despite assurances regarding confidentiality and anonymity, there were many instances where respondents who fit the inclusion criteria chose not to be involved through fear of reprisals from out of town drug dealers.^3^ Moreover, qualitative research with vulnerable people who have been subject to various forms of abuse reports a range of inhibitions to disclosing information including mistrust of others,^34^ fear of not being believed ^35^ and fear of perpetrator retribution.^34^, ^36^ Certainly, in this work, the reluctance to be seen as a ‘grass’ appeared responsible for many refusals to participate. The adage ‘snitches get stitches’ was often cited. Efforts were made to build trusting relationships, with a particular focus on establishing clear communication channels with messages about the research being repeated and in plain language. Strict adherence was paid to confidentiality and it was stressed that the YP was in control of all subject matter touched on during the sessions.

### 4.3 Insider/outsider status, reflexivity and power

The researcher who conducted the interviews for this piece of work (R. B.) is a white woman of 60 years who acknowledges that her biases, assumptions, values, experiences and position of power are likely to be fundamentally different to those of the research participants and it is important to reflect on how this has impacted on the research, both in terms of the questions being asked, the answers given and our interpretation of the data. Importantly, in the interests of transparency and reflexivity, R. B. did not embark on this research from a neutral standpoint but has personal involvement of a family member who became actively involved, from the age of 14, in a County Lines drug gang. She has been witness to and is familiar with many of the painful stories told by the research participants in their progression into violent gang culture. Indeed, this study was conceived as a way to improve our understanding of the perspective of the marginalised child, the child who finds mainstream society a hard place to flourish. For these reasons, she makes no pretence of being impartial but, rather, embarked on this work expressly to gather the stories of this vulnerable group of YP, as told from their own mouths, with the hope that in providing them with a means to get their voice heard and needs better met.

This life experience of the researcher was something shared with participants before the interview session, and often meant that doors which were previously closed were then held open. There was an apparent transformation in perception from ‘professional with clip‐board status’ to someone who had valid reasons to hear about their lived truth. In the language of qualitative ethnographic research, she was given the way in as an ‘insider’. Debates continue to rage about the relative benefits of ‘insider’ versus ‘outsider’ qualitative research practice. Some call for a balance of ‘involvement and estrangement’—the benefits of being familiar with culture and the issues under discussion are weighed against the dangers of being too partial or over‐involved.^37^ ^,p.485^ Others argue that all knowledge is partial and must be made up of multiple perspectives and viewpoints.^38^ During the planning phase for this project members of the advisory committee suggested that interviews should be conducted by those with closer match (both in terms of demographics and experience) and while this was considered, the logistics within the resources available proved prohibitive. In retrospect, it is evident that had we adopted closer demographic matching (between interviewer and interviewee), particularly on the basis of ethnicity, materially different outcomes would have resulted. We suggest that no outcome would be more or less valid—simply a different piece of the puzzle provided, contributing towards a more complete understanding. Our intention has been to acknowledge our biases with the ambition to provide, as far as possible, an accurate representation of one aspect of the social world under study.

Research of this nature is built on establishing trusting relationships, influenced by demographics (race, gender, class) as well as personal characteristics. While some argue that ethnic matching builds possibilities of a ‘safe place’ that maximises rapport and trust, others suggest that, when considering sensitive issues, there are advantages when the interviewer is viewed as neutral or ‘other’.^39^ Yet, the intersections between race, class, gender and culture are complex, and the understanding of ‘otherness’ is increasingly called into question. The basis, therefore, by which interviewer and interviewee should be matched is open to multiple views and interpretations.

The methodological approach taken and the points we have drawn out of the research are shaped by culturally driven and value laden ontological assumptions.^40^ Moreover, interviews of this type can't but have an emotional pull. Many of the accounts presented by YP were of such a harrowing and brutal nature that the process inevitably involves a degree of human empathy and reaching out. Qualitative research can never be objective though a reflexive lens helps acknowledge the role of values and the tendency to ‘take sides’.^41^

### 4.4 Reflections on the interview process

The tempo and feel of interviews varied, some starting slowly with YP initially offering monosyllabic answers, gradually warming up as they realised that there were no right or wrong answers, and recognising that their own narrative was central to the process. While there were a couple of characters who remained reticent and only marginally engaged, the majority appeared to reflect openly on their own situations. At regular points in the interview, the researcher checked in to ask how they were finding the process and to see if they wanted to stop. A small number found it hard to be reflexive and mentioned that the medication they were taking for attention deficit and hyperactivity disorder made them feel a bit flat or that their current circumstances were getting them down. Most however appeared to enjoy the session, noting that it was ‘good to be listened to’. One YP, interviewed in an alternative provision, initially had said he didn't want to participate but was encouraged by the unit manager, and visibly warmed up through the course of the session. Despite admitting to feeling anxious about our session; ‘you can tell I'm anxious by the way I'm sitting’, he pushed himself to do it.

I like explaining myself to people when I get the chance. Most of my life I haven't had the chance to talk to anyone. No one really listens to you when you grow up but I want to be understood…

And then later on in the interview

Interviewer: How are you doing F?

F: I'm doing fine. Like I say… It doesn't matter if it's for one second or one minute or one hour… I'll speak to someone for however long they want as long as I enjoy talking to them.

In another case, in a youth club in South West London, a young person involved in local drug dealing, with a father imprisoned throughout his childhood, described himself as a ‘good G in a bad place’ and agreed to the interview when he understood the motivation was to better understand the perspective of the YP. Immersed in violent ‘gang land’ culture, he was nervous initially about being recorded and constantly sought assurance that he wasn't telling me anything ‘incriminating’. He claimed to find it hard just to talk and encouraged me to ask questions:

You need to ask me specific questions. Me I'm not good at just talking… If I find a question a bit hard to answer I'll just tell you.

Yet, despite his anxieties, T was extraordinarily reflective and at the end of the interview, when offered his £40 JD voucher as a thanks, shrugged his shoulders muttering that JD wasn't his ‘place’ and offered the voucher to another pair of eager hands.

In summary, the key ways in which we worked to access marginalised YP and build an environment in which they felt enabled to share experiences included:

1. Taking time to build trust and convey understanding of the project.
2. Consideration of the different approaches adopted by statutory organisations and smaller voluntary/charitable groups who may have different levels of tolerance for risk.
3. Being open about the interviewer's personal context as an ‘insider’.^42^
4. Working with the ethics committee in relation to requirements for parental consent.
5. Consideration of power dynamics.
6. Maintaining flexibility in my research methods and trialling methods to build a nonthreatening environment—for example, through using creative activities as part of the interview process.

## 5 LIMITATIONS TO METHODS USED

This research was necessarily pragmatic. Using gatekeepers to access YP creates bias in the sample, with those interviewed likely to have better social skills and relationships with peers and associated professionals than some of their peers.^4^ Moreover, although the research set out to interview both males and females, only 2 of the total sample of 29 were girls. Given the different profiles and characteristics of gang‐involved girls and boys, further consideration is needed to develop research methods which are appropriate for use with gang‐involved girls.

## 6 CONCLUSION

In this age of ubiquitous social media, where the views and opinions of YP are often hidden from older generations in digital posts, behind encrypted messaging systems, there is more onus on the research community to find ways to engage YP as partners in research.^43^ Researchers have reported a tendency to avoid conducting research with YP under the age of 18 years, being put off by the rigours of the ethical procedures they will need to contend with‐ yet talking to YP in this research has heightened our awareness of the richness of the data that can be collected, the value YP attach to being listened to and the importance of seeing through the process, however arduous it might seem.

While the work reported here falls short of following truly participatory research methods which aim to involve researchers and researched as equal partners in setting the research agenda, developing methods, collecting and disseminating data^44^—we contend that in striving to access marginalised YP and in seeking greater diversity and inclusion we created a culture which facilitated meaningful participation. Child‐friendly techniques do not, in themselves, equate to ‘the equalisation of power relationships’.^11^ YP who participated in this research had different preferences for self‐expression and responded to the interview techniques employed in a variety of distinct ways. Being sensitive to individual preferences for communication, allowing multiple means of expression (drawing, timelines, narratives, questions) and triangulating data through observations and data supplied by linked professionals, helped validate and enrich individual accounts. Methods used were strongly influenced by initial PPI sessions in which techniques were tested with members of our target group, yet there was a degree of pragmatism and iterative practice with adjustments and modifications made as the need arose.

Mental health services for YP, particularly postpandemic, are facing a significant rise in demand. Mental health problems are often manifested through externalising antisocial behaviours leading to school exclusion and/or criminal convictions.^45^ Finding ways to access the seldom‐heard voices of this vulnerable population should help to ensure that mental health and other support services are more appropriately designed and responsive to their needs.^45^ In this way, appropriate therapeutic services may replace a more reactive, punitive response. The rich narratives emerging from this work are being used to inform mental health and education policies– particularly in relation to the impact of current service structure on the most marginalised populations. To improve the rigour of evidence and build on some of the methodological challenges discussed here, we suggest that further methodological studies are needed to look at undertaking longitudinal research with disadvantaged populations.

Our intention, in laying out some of the ethical and methodological challenges confronted in interviewing vulnerable YP, has been both to offer practical advice and guidance that can help inform future work with seldom heard populations.

## AUTHOR CONTRIBUTIONS

**Rhiannon Barker**: Conceptualisation; investigation; funding acquisition; writing—original draft; methodology; validation; visualisation; formal analysis; project administration; data curation. **Chris Bonell**: Supervision; writing—review and editing. **G. J. Melendez‐Torres**: Writing—review and editing; supervision.

## CONFLICT OF INTEREST STATEMENT

The authors declare no conflict of interest.

## ETHICS STATEMENT

Ethics permission was received from the London School of Hygiene and Tropical Medicine ethics committee in November 2022 (reference: 26873). Written and oral consent was collected from all research participants.

## Data Availability

Anonymised data that support the findings of this study are available on reasonable request from the corresponding author, Rhiannon Barker. The data are not publicly available due to containing information that could compromise the privacy of research participants.

## APPENDIX A

**Table A1:** Demographic summary of interviews with young people (YP).

Interviews with YP (*n *= 29)	
Age	14 years–3
	15 years–13
	16 years–2
	17 years–5
	18 years–1
	19 years–2
	20 years–2
	24 years–1
Ethnicity	White British–18
	Black African–1
	Dual Heritage–5
	Caribbean–3
	Armenian–1
	Pakistani–1
School exclusion	26 (mainly permanent)
Criminal involvement (recorded if YP reported they had been arrested/held by the police)	24
Links to criminal gangs (recorded if gang involvement was acknowledged by participants)	13
Mental health diagnosis (recorded if YP had formal diagnosis or referral to child and adolescent mental health services)	15
Interview settings	Alternative provision, youth offending teams, late night youth club, charities supporting gang‐involved YP
Geographic locations	Liverpool, London, West Country
